# Hypoxia modulates human eosinophil function

**DOI:** 10.1186/1476-7961-8-10

**Published:** 2010-07-19

**Authors:** Alon H Nissim Ben Efraim, Ron Eliashar, Francesca Levi-Schaffer

**Affiliations:** 1 Department of Pharmacology and Experimental Therapeutics, Institute for Drug Research, School of Pharmacy, Faculty of Medicine, The Hebrew University of Jerusalem, Jerusalem, Israel; 2 Department of Otolaryngology/Head and Neck Surgery, Hadassah Hebrew University Medical Center, Jerusalem, Israel

## Abstract

**Background:**

Eosinophils are involved in various inflammatory processes including allergic inflammation during which angiogenesis has been documented. Angiogenesis is most likely connected to the hypoxia which characterizes inflamed tissues. Eosinophils produce VEGF and are pro-angiogenic. However, to the best of our knowledge no study has been performed to verify the existence of a direct link between eosinophils, hypoxia and angiogenesis in allergic inflammation.

**Objective:**

To characterize eosinophil function and angiogenic potential under hypoxic conditions.

**Methods:**

Human peripheral blood eosinophils were cultured in normoxic or hypoxic conditions with or without cytokines. Viability and apoptosis were assessed by Annexin V/PI staining. Anti- or pro-apoptotic protein levels, HIF-1α levels and MAPK phosphorylation were analyzed by immunoblot analysis. Angiogenic mediator release was evaluated by ELISA.

**Results:**

Hypoxic eosinophils were more viable than normoxic ones after up to three days. In addition in hypoxia, anti-apoptotic Bcl-XL protein levels increased more than pro-apoptotic Bax levels. Hypoxia increased VEGF and IL-8 release. In hypoxic eosinophils high levels of HIF-1α were observed, particularly in the presence of GM-CSF. MAPK, particularly ERK1/2 inhibitors, decreased hypoxia-mediated VEGF release and HIF-1α expression.

**Conclusion:**

Eosinophils respond to hypoxia by up-regulation of survival and of some of their pro-angiogenic functions indicating a correlation between eosinophilic inflammation and angiogenesis.

## Introduction

Allergic diseases are generally characterized by inflammation, in which tissue infiltration of myeloid cells, mainly eosinophils and Th2 cells, is an important feature [1-5].

The microenvironment of injured inflamed tissues is mostly characterized by high concentrations of lactate and reductive metabolites, as well as by low levels of glucose and oxygen [6]. This low oxygen level, or hypoxia, is due to an inadequate blood supply and high consumption of oxygen by the infiltrated cells [7-9].

Hypoxic conditions have been shown to profoundly affect a broad range of myeloid cell properties *in vitro*, e.g., phagocytosis, cell surface marker expression, secretion of cytokines, chemokine receptor levels, adhesion, migration, and cell survival [9]. In addition, hypoxia promotes remodeling and angiogenesis, the sprouting of new blood vessels from pre-existing ones, thus renewing the blood supply and increasing oxygen levels in the tissue [10,11]. Several transcription factors are involved in the response to hypoxic stress. Among them, hypoxia inducible factor (HIF)-1 functions as a master regulator of oxygen homeostasis and is responsible for vascular endothelial growth factor (VEGF) synthesis [9,12]. Interestingly, in asthmatic lungs as well as in nasal polyps, there is a high expression of VEGF [8,13-16].

Considering the key function of eosinophils as effector cells in allergic inflammation, we became interested in their role in angiogenesis. The first important link between eosinophils and angiogenesis was reported by Horiuchi et al. [17]. They demonstrated that eosinophils contain VEGF protein in their granules and release it after stimulation with either granulocyte macrophage colony stimulating factor (GM-CSF) or interleukin (IL)-5.

We have shown that eosinophils display a direct pro-angiogenic effect promoting endothelial cell proliferation, inducing VEGF production by endothelial cells, and rendering these cells more sensitive to VEGF via up-regulation of the VEGF receptor [18]. These phenomena appear to be mostly, but not exclusively, mediated by VEGF. Recently we demonstrated that the eosinophil cationic major basic protein (MBP) can induce angiogenesis in the same experimental models [19]. Moreover, we found that eosinophils express osteopontin, a glycoprotein molecule which exhibits pro-fibrogenic and pro-angiogenic properties and is implicated in allergic diseases [20].

To the best of our knowledge, there are no reports on the influence of hypoxia, the main driver of angiogenesis, on eosinophil functions. In this study we therefore aimed to investigate the possible effect of hypoxia on eosinophil activity and pro-angiogenic potential.

## Materials and methods

### Cells

Eosinophils were purified as previously described [21,22] from the peripheral blood of untreated, mildly atopic volunteers (blood eosinophil levels 5%-10%), who were asymptomatic and therefore not taking any drug for their condition, and on the day of blood donating, any other drug. Written informed consent was obtained according to the guidelines of the Hadassah-Hebrew University Human Experimentation Helsinki Committee. Briefly, venous blood (150 ml) was collected in heparinized syringes and left to sediment in 6% dextran (Sigma Chemicals, St Louis, MO). Leukocytes were centrifuged on Ficoll-Hypaque (density 1.077; Sigma Chemicals) for 25 minutes at 700 *g *at 20°C. Neutrophils and lymphocytes were tagged in the granulocyte-enriched pellet with micromagnetic beads bound to anti-CD16 and anti-CD3 antibodies, respectively (Miltenyi Biotech GmbH, Bergisch Gladbach, Germany). Eosinophils were purified by passing this cell suspension through a magnetic field and were then collected at a purity of >98% (Kimura staining), with a viability of >98% (trypan blue staining). Thereafter, eosinophils were re-suspended (5*10^6 ^or 10^6 ^cells/ml) in culture medium consisting of RPMI 1640 supplemented with L-glutamine (300 mg/l), 10% heat-inactivated FCS, and penicillin-streptomycin solution (100 u/ml) (Biological Industries, Beit Haemek, Israel).

### Eosinophil culture in hypoxic or normoxic condition

Eosinophils were cultured in 96 well u-shaped plates (Nunc, Rochester, NY) in 200 μl of medium alone or supplemented with cytokines (see below) according to the experiment. For experiments in hypoxia, plates were placed in a closed humidified chamber at 37°C, with a continuous flow of gas mixture of 95% N_2 _and 5% CO_2_. The oxygen percentage in the medium was monitored with a dissolved oxygen meter before each time point checked and found to be ≤3% (MettlerToledo, Schwerzenbach, Switzerland). Two hours after the starting of gas flow in the chamber, the oxygen level equilibrated at <3%. At this time point eosinophils were added to the wells. All the assays were performed on eosinophils cultured for a further 1 h, to permit full re-equilibration of the oxygen in the chamber (<3%). For experiments in normoxia, plates were placed at 37°C in a humidified incubator with 5% CO_2 _and air.

### Annexin V and propidium iodide (PI) staining and flow cytometry

Eosinophils (10^6 ^cells/ml, 0.2 ml) were incubated either in normoxia or hypoxia in the presence or absence of GM-CSF, IL-3, or IL-5 (20 ng/ml; Peprotech, Rocky Hill, NJ) for 24, 48 and 72 h. Cells were then washed and re-suspended in 0.2 ml buffer (218 mM Hepes, 1.4 mM sodium chloride, 38.1 mM calcium chloride) and incubated for 20 min on ice with AnnexinV-FITC (10 μl/10^6 ^cells; IQ Products, Groningen, Netherland). The cells were washed and propidium iodide (5 μg/ml) was added. Apoptosis and viability were analyzed by flow cytometry (FACScalibur System, Becton Dickinson, San Jose, CA).

### HIF-α staining for flow cytometry

Eosinophils (10^6 ^cells/ml, 0.2 ml) incubated for the indicated time points in medium in either normoxia or hypoxia, were fixed in 2% formaldehyde (15 min, 4°C), washed in a final volume of 100 μl of HBSS supplemented with 0.1% BSA and 0.02% sodium azide (HBA) for 30 min on ice and permeabilized in HBA containing Saponin 0.1%, BSA 10%, serum 0.1%, and Hepes 10 mM (20 min, RT). Anti- HIF-1α (5 μg/ml; R&D Systems, Minneapolis, MN) or irrelevant control Abs were added to these fixed permeabilized cells (30 min on ice), and cultures were incubated with anti-goat Cy5-conjugated IgG (1:100; 20 min, RT; Jackson ImmunoResearch Laboratories, West Grove, PA). After staining, the cells were analyzed by flow cytometry collecting 10,000 events, and data analysis was performed using CellQuest software (BD Biosciences).

### Immunoblot determination of eosinophil protein expression

For MAPK analysis, eosinophils (5 × 10^6^cells/ml, 0.2 ml) were incubated in normoxia or hypoxia for 1 h and 3 h in medium alone, or in medium containing GM-CSF with or without the specific inhibitors for ERK1/2 (PD98059) or p38 (SB203580; 10 μM; Calbiochem, San Diego, CA). The inhibitors were added to the media at the beginning of the culture. After incubation, cells were immediately centrifuged (3 min, 4°C 150 g) and re-suspended in sample buffer X1.5 (10^6 ^cells in 50 μl), boiled for 10 min, and vortexed. Cells were counted before incubation and in representative experiments after incubation, and no significant differences in cell numbers were found. Samples (15 μl) were resolved in SDS-PAGE using 10% polyacrylamide gel and transferred to nitrocellulose membranes. Membranes were incubated for 1 h at RT with anti-ERK1/2 (1:1000; Cell Signaling Technology, Danvers, MA), anti-phospho ERK1/2 (1:1000; Cell Signaling Technology), anti-pP38 (1:1000; BD Bioscience, San Diego, CA) antibodies, and then for 1 h with horseradish peroxidase (HRP)-conjugated goat-anti-mouse or goat-anti-rabbit antibodies (1:10000; Jackson ImmunoResearch Laboratories). For HIF-1α analysis samples were loaded in 8% SDS-PAGE gel and incubated o.n. with goat anti-HIF-1α antibodies (1:1000; R&D System) and then with HRP-conjugated anti-goat antibodies (1:10000; Santa Cruz Biotechnology, Santa Cruz, CA) for 1 h. For apoptotic protein analysis, samples were loaded in 15% SDS-PAGE gel. Membranes were incubated with goat anti-Bax or anti-Bcl-XL antibodies (1:1000; Cell Signaling Technology) and with HRP-conjugated anti-goat antibodies (1:10000; Cell Signaling Technology). Detections were made by ECLplus (GE Healthcare, Amersham, UK).

### ELISA

Eosinophils [5 × 10^6 ^cells/ml, 0.2 ml] were cultured in normoxic or hypoxic conditions o.n. and supernatants were collected after centrifugation. VEGF was detected by Human VEGF ELISA Development kit (PeproTech Inc.) and IL-8 by DuoSet kit (R&D system) according to the manufacturer's instructions. The lower limit of the assay sensitivity was 16 pg/ml in both cases.

### Statistical analysis

The control and experimental groups were compared by paired *t*-test for evaluation of significance (p < 0.05). The data are expressed as mean ± standard error of measurement (SEM) of at least three independent experiments performed in triplicate. The KyPlot™ analysis tool-pack was used to perform the statistical analysis.

## Results

### Hypoxia increases peripheral blood eosinophil survival

**Figure 1:**
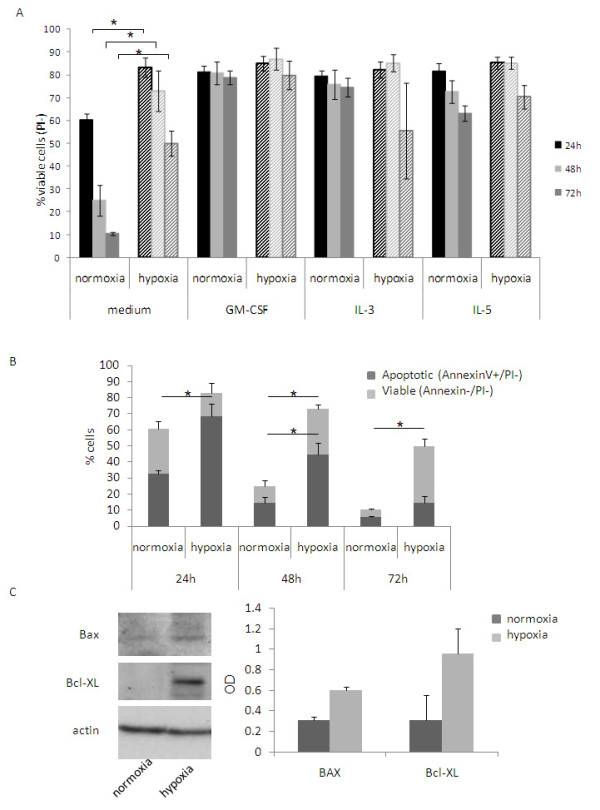
**Effect of hypoxia on eosinophil survival**. Eosinophils were cultured in medium alone or supplemented with either GM-CSF, IL-3 or IL-5 (20 ng/ml) in normoxia (solid columns) or hypoxia (stripped columns) for 24 h, 48 h and 72 h. (A) Flow cytometry analysis of PI stained cells was performed and viable eosinophils were identified as PI^-^. (B) Eosinophils were cultured in medium in hypoxia or normoxia for 24 h, 48 h and 72 h and stained with AnnexinV and PI. AnnexinV^+^/PI^- ^cells were gated as viable apoptotic eosinophils; AnnexinV^-^/PI^- ^were gated as viable cells. (C) Eosinophils were cultured o.n. in medium in normoxia or hypoxia and Bax and Bcl-XL expression was analyzed by Western blot after o.n. incubation (C). Representative blot of three experiments performed on three different eosinophil donors (left panel), and average of densitometry quantification of the three experiments (right panel) are shown.

To determine whether hypoxic conditions influence eosinophil behavior, eosinophils were cultured in hypoxia or in normoxia in medium alone, or with an optimal concentration of either GM-CSF, IL-3, or IL-5. After 24 h in medium, the percentage of viable eosinophils in hypoxia was significantly higher than in normoxia (83.1 ± 4.21% in hypoxia and 60.42 ± 2.63% in normoxia, Fig. 1A; p = 0.019). Similar results were obtained after 48 h of culture (72.8 ± 8.93% and 24.98 ± 6.8% respectively; p = 0.013) and were more pronounced after 72 h (49.96 ± 5.41% and 10.46 ± 0.74%, p = 0.018). The viability of eosinophils cultured with either one of the three survival cytokines was ~80% for normoxia and hypoxia at all three time points assessed.

The impact of hypoxia on eosinophil survival was evaluated by specifically assessing their viability/apoptosis using AnnexinV/PI double staining. After 24 h in normoxia, PI negative eosinophils were ~60% and 28.03 ± 4.95% were AnnexinV positive. In the case of hypoxic eosinophils, ~80% were PI negative cells and only 14.47 ± 6.13% were AnnexinV positive (p = 0.043). Also after 48 h there were significant differences between normoxic and hypoxic AnnexinV positive eosinophils (normoxia 10.3 ± 3.68%, hypoxia 28.28 ± 2.67%; p = 0.037). After 72 h the differences were no longer significant (normoxia 4.76 ± 0.64%; hypoxia 35.34 ± 4.42%). To further investigate the involvement of apoptosis in the death of eosinophils, Bax and Bcl-XL, pro- and anti-apoptotic proteins respectively, were analyzed by Western blot in normoxic and hypoxic eosinophils. After o.n. incubation, normoxic eosinophils expressed similar levels of BAX and Bcl-XL. In hypoxic conditions Bcl-XL expression was strongly increased while that of Bax slightly augmented (Fig. 1C).

### Hypoxia increases VEGF and IL-8 release from eosinophils

**Figure 2:**
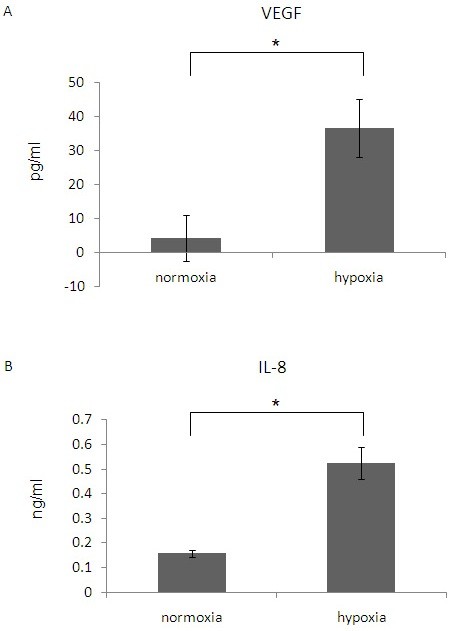
**The effect of hypoxia on VEGF and IL-8 release from eosinophils**. Eosinophils were cultured in medium in normoxia or hypoxia. After o.n. incubation VEGF (A) and IL-8 (B) concentrations were measured in supernatants by specific ELISAs. Data are the mean ± SEM of three experiments. (* p ≤ 0.05; ** p ≤ 0.005).

To study the influence of hypoxia on eosinophil activity, the release of different factors was evaluated. Hypoxia did not influence eosinophil degranulation as assessed by EPO release after 1 h of incubation (data not shown). However, eosinophils cultured o.n. in hypoxic conditions released significantly more VEGF compared to cells cultured in normoxic conditions (Fig. 2A; normoxia: not detectable; hypoxia: 60.03 ± 17.87 pg/ml; p = 0.0186). In addition, IL-8 release was significantly higher in hypoxic eosinophils (2639.17 ± 258.15 pg/ml) than in normoxic ones (831 ± 52.1 pg/ml) (Fig. 2B; p = 0.005).

### Hypoxia increases HIF-1α levels in eosinophils

**Figure 3:**
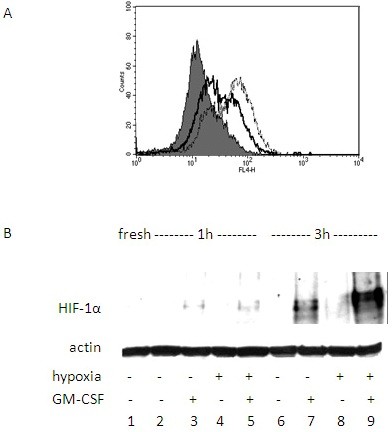
**HIF-1α expression by eosinophils**. (A) Flow cytometry analysis of intracellular staining for HIF-1α in eosinophils incubated in normoxia (solid line) or hypoxia (dashed line) up to 3 h. (B) Western blot analysis for HIF-1α levels in eosinophils incubated in normoxia or hypoxia with or without GM-CSF for 3 h. Data are representative of three experiments.

We then evaluated whether hypoxia affects HIF-1α levels in eosinophils. HIF-1α expression was assessed after 1 or 3 hours, in order to evaluate the short-term hypoxia-mediated up-regulation of this factor due to its stabilization, rather than to synthesis of new proteins, as in longer time points. Flow cytometric analysis for intracellular staining of eosinophils showed an increase in the MFI of HIF-1α after 3 h in hypoxia compared with normoxia (Fig. 3A). This hypoxic dependent increase in HIF-1α was confirmed by Western blot analysis of eosinophils cultured 1 h and 3 h in medium alone or in the presence of GM-CSF (Fig. 3B). After 1 h, no evident difference in HIF-1α expression was detected between normoxia and hypoxia (lanes 2 and 4). At this time point the cells incubated with GM-CSF showed a faint band for HIF-1α in both normoxia and hypoxia (lanes 3 an 5). After 3 h, eosinophils cultured in medium displayed no band in normoxia (lane 6) but a faint band was still present in hypoxia (lane 8). At this time point in the presence of GM-CSF, HIF-1α was evident in normoxia (lane7) and a stronger signal was detected in hypoxia (lane 9).

### Effect of GM-CSF on hypoxia-dependent VEGF release and HIF-1α regulation in eosinophils

Because of the GM-CSF enhancing effect detected above, we incubated eosinophils in the presence of GM-CSF under normoxic and hypoxic conditions and evaluated VEGF release. Hypoxia in the presence of GM-CSF induced higher release of VEGF (186.46 ± 33.93 pg/ml) when compared to the normoxic levels (73.46 ± 36.15 pg/ml). In the case of IL-8, the presence of GM-CSF did not influence the cytokine release (data not shown).

**Figure 4:**
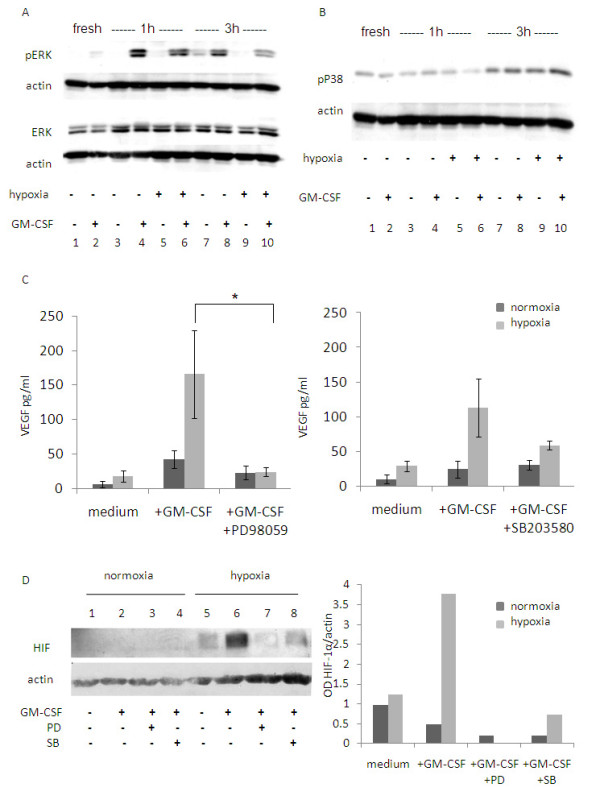
**Effect of GM-CSF signaling on hypoxia mediated HIF-1α and VEGF regulation**. Eosinophils were incubated in normoxia/hypoxia in the absence or presence of GM-CSF. Western blot analysis was performed for phospho ERK1/2 and total ERK1/2 (A) or pP38 (B). The results are from one representative experiment out of three. (C) Eosinophils were cultured in medium with or without GM-CSF and with or without PD98059 (left panel; n = 5) or SB203580 (right panel; n = 3). MAPK inhibitors were added at the starting of the culture. VEGF levels were analyzed by ELISA. Data are the mean ± SEM of three experiments (* p ≤ 0.05). (D) Eosinophils were cultured for 3 h in medium alone (lanes 1 and 5), with GM-CSF alone (lanes 2 and 6) or with GM-CSF with PD98059 (lanes 3 and 7) or SB203580 (lanes 4 and 8). HIF-1α levels were analyzed by Western blot (left panel). Densitometry analysis is shown as a column chart (right panel). Data are representative of four experiments.

In the next series of experiments we therefore analyzed the effect of MAPK phosphorylation as a down-stream signaling of GM-CSF. ERK1/2 and p38 MAPK phosphorylation were evaluated in normoxia/hypoxia. After 1 h and 3 h of incubation no bands were detected when eosinophils were cultured in medium alone (M.W. 42 KDa; Fig. 4A lanes 3 and 5 and lanes 7 and 9). However, when incubated with GM-CSF, eosinophils displayed two bands corresponding to pERK1/2 both in normoxia and in hypoxia after 1 h (lanes 4 and 6) and after 3 h (lanes 8 and 10). The expression of the total ERK1/2 was similar in all samples. When the phosphorylated p38 was analyzed, no differences between normoxia and hypoxia were observed (Fig. 4B; lanes 3 and 5). In this case, addition of GM-CSF had no evident effect on p38 phosphorylation (lanes 4 and 6). A similar result was obtained after 3 h of incubation (lanes 7 and 9 and lanes 8 and 10).

When PD98059 was added together with GM-CSF in o.n. cultures, a significant decrease in VEGF release under hypoxic conditions was observed compared to cultures with no inhibitor (Fig. 4C; hypoxia: with GM-CSF 165.62 ± 63.83 pg/ml; with GM-CSF and PD98059 23.77 ± 6.18 pg/ml; p < 0.05; normoxia: with GM-CSF 42 ± 12.7 pg/ml; with GM-CSF and PD98059 22.9 ± 9.97 pg/ml; n.s.). In addition, when the p38 MAPK inhibitor SB203580 was added to the GM-CSF cultured eosinophils in hypoxia, VEGF release decreased, though not significantly (Fig. 4C; normoxia: with GM-CSF 24.34 ± 12.13 pg/ml; with GM-CSF and SB203580 30.64 ± 7.25 pg/ml; hypoxia: with GM-CSF 113.26 ± 41.93 pg/ml; with GM-CSF and SB203580 58.55 ± 6.33 pg/ml; p < 0.05).

The influence of MAPK inhibitors was also tested on HIF-1α regulation. As shown in Fig. 4D, in 3 h hypoxic cultures in the presence of GM-CSF, HIF-1α levels highly decreased and reached levels similar to those in normoxia when PD98059 was added (lane 7). Addition of SB203580 slightly decreased HIF-1α expression in these hypoxic cultures (lane 8).

## Discussion

Inflammation, angiogenesis, and remodeling are present in allergic inflamed tissues together with prominent eosinophil infiltration. We hypothesized that the inflamed hypoxic milieu can lead to eosinophil activation and promote neo-vascularization in allergic states, and investigated whether hypoxia could influence eosinophil properties *in vitro*.

First, we found that hypoxia increases the viability of eosinophils up to three days. This result confirms a previous work in which hypoxic conditions were found to prolong eosinophil viability, as measured by trypan blue exclusion assay [23]. We further evaluated the influence of hypoxia on eosinophil apoptosis and found an increase in both PI^-^/AnnexinV^- ^and PI^-^/AnnexinV^+ ^cells in hypoxia. This would suggest a delaying effect on the eosinophil apoptotic pathway. In addition, the increase of the anti-apoptotic protein Bcl-XL more than the pro-apoptotic protein Bax in hypoxia, confirmed that this survival can be partially due to the inhibition of apoptosis. Interestingly, no differences in GM-CSF release were observed in eosinophils cultured in normoxia vs. hypoxia (data not shown). This data would suggest that the increased viability of the cells is not a consequence of increased GM-CSF production by hypoxic eosinophils.

Second, we found that hypoxia increases the release of the most important pro-angiogenic factor VEGF from eosinophils. VEGF is the key mediator of angiogenesis, being a specific mitogen for endothelial cells [24,25]. Many cells, including structural cells such as endothelial cells and inflammatory cells, release VEGF as a response to hypoxia [9,11,26]. Interestingly, this effect was enhanced by the presence of GM-CSF in a seemingly synergistic fashion. In addition, the release of IL-8, which stimulates endothelial cell proliferation and capillary tube organization [27], was also found to be augmented in eosinophils under hypoxia. It is important to point out that in the time point in which cytokine release was evaluated, no significant differences in cell viability were observed (data not shown).

Finally, we demonstrated for the first time that eosinophils express HIF-1, and that its subunit HIF-1α is stabilized after a short exposure to hypoxic conditions in these cells. Based on the literature on HIF-1α induction, it may be concluded that the classical stabilization mechanism induced by hypoxia causes the up-regulation of HIF. In addition in our study, HIF-1α up-regulation was further enhanced in the presence of GM-CSF. This effect of GM-CSF seems to correlate to its increasing effect on VEGF release. In assays performed after o.n. incubation, HIF-1α levels were found to be still high (data not shown).

In eosinophils, GM-CSF-induced ERK1/2 phosphorylation is involved in their migration and degranulation [28,29]. MAPK cascade is known to participate in cell proliferation, differentiation, survival, and locomotion [30]. In addition, ERK1/2 has a specific role in regulating VEGF gene expression [31,32]. Richard et al. showed in fibroblasts that HIF-1α is strongly phosphorylated by ERK1/2 and that this is sufficient to promote the transcriptional activity of HIF-1 on VEGF [32]. In the current study we showed that inhibition of ERK1/2 phosphorylation decreased both GM-CSF-induced HIF-1α and VEGF up-regulation in eosinophils. Interestingly, although no effect of hypoxia on ERK1/2 phosphorylation could be detected, PD98059 addition decreased both ERK1/2 and VEGF in eosinophils, to levels comparable to normoxia. This may be due to the multiple roles and interactions of ERK signaling.

In summary, GM-CSF increases HIF-1α levels and VEGF release by inducing ERK1/2 phosphorylation in a hypoxia-independent way. We suggest that GM-CSF might render the eosinophils more responsive to hypoxia-induced activation by enhancing MAPK phosphorylation, which in turn contributes to HIF-1 stabilization.

The present data showing cooperation between hypoxia and GM-CSF on eosinophil function might be very relevant to what happens *in vivo *in allergic inflamed tissues, in which high levels of GM-CSF and various levels of hypoxia frequently coexist [33-35]. We believe that because of their high infiltration in chronic allergic inflammation, eosinophils play a pivotal role in promoting angiogenesis in these tissues. As other inflammatory, as well as structural cells, are involved in tissue response to hypoxia, further studies should assess the relative contribution of the eosinophils to this phenomenon.

## Abbreviations

ERK: Externally regulated kinase; BAX: Bcl-2-associated X protein; Bcl: B-cell lymphoma; GM-CSF: Granulocyte macrophage- colony stimulating factor; HIF: Hypoxia inducible factor; IL: Interleukine; MAPK: Mitogen-activated protein kinase; VEGF: Vascular endothelial growth factor

## Competing interests

The authors declare that they have no competing interests.

## Authors' contributions

AN carried out the immunoblots, the flow cytometry and the ELISAs, performed the statistical analyses and drafted the manuscript. RE participated in the design of the study and supplied donor's samples. FLS conceived of the study and participated in its design and coordination. All authors read and approved the final manuscript.
